# A Phase II Randomized Controlled Trial Comparing Safety, Procedure Time, and Cost of the PrePex™ Device to Forceps Guided Surgical Circumcision in Zimbabwe

**DOI:** 10.1371/journal.pone.0156220

**Published:** 2016-05-26

**Authors:** Mufuta Tshimanga, Tonderayi Mangwiro, Owen Mugurungi, Sinokuthemba Xaba, Munyaradzi Murwira, Danuta Kasprzyk, Daniel E. Montaño, Daisy Nyamukapa, Basile Tambashe, Pesanai Chatikobo, Patricia Gundidza, Gerald Gwinji

**Affiliations:** 1 Department of Community Medicine, College of Health Sciences, University of Zimbabwe, Harare, Zimbabwe; 2 Department of Surgery, College of Health Sciences, University of Zimbabwe, Harare, Zimbabwe; 3 Ministry of Health and Child Care, Harare, Zimbabwe; 4 Zimbabwe National Family Planning Council, Harare, Zimbabwe; 5 Battelle Health and Analytics, Seattle, United States of America; 6 United Nations Population Fund, Harare, Zimbabwe; Johns Hopkins University Bloomberg School of Public Health, UNITED STATES

## Abstract

**Background:**

The World Health Organization (WHO) and the Joint United Nations Program on HIV/AIDS promote MC (male circumcision) as a key HIV prevention strategy where HIV prevalence and incidence are high and MC prevalence is low. In Zimbabwe, to achieve the 1.26 million circumcisions needed to be performed by 2015 to achieve optimal MC coverage, a new approach was needed. The primary objective of the current trial was to assess the performance (safety, procedure time, and cost) of the PrePex device compared to forceps-guided surgical circumcision.

**Methods and Findings:**

This Phase II, randomized, open-label trial in Zimbabwe involved healthy, non-circumcised adult male volunteers who were randomly assigned to the PrePex device (n = 160) or surgical arm (n = 80). Three doctors and 4 nurses, all certified on both circumcision methods, performed the procedures. The PrePex device procedure involves a plastic ring with a rubber O-ring that necrotizes the foreskin to facilitate easy and minimally invasive removal. Total procedure time was the primary endpoint. Adverse event (AE) data were also gathered for 90 days post-procedure. All 80 participants in the surgical arm and 158 participants in the PrePex arm achieved complete circumcision. The total procedure time for the PrePex device was approximately one-third of the total surgical procedure (4.8 minutes, Standard Deviation [SD]: 1.2 versus 14.6 minutes; SD: 4.2; p<0.00001). There were 2 AEs for 2 participants (rate of 1.3%, 95% Confidence Interval: 0.0025–4.53%), which were resolved with simple intervention. The AEs were device related, including 1 case of pain leading to device removal and 1 case of removal of the device.

**Conclusions:**

The trial supports previous studies’ conclusions that the PrePex procedure is safe, quick, easy to apply, and effective in terms of procedure time as an alternative to traditional surgical circumcision. The PrePex device has great potential for use in overburdened health systems and in resource-limited settings and is recommended for use in rapid scale-up of adult MC in Zimbabwe.

**Trial Registration:**

ClinicalTrials.gov NCT01956370

## Introduction

Accruing evidence of meta-analyses and 3 randomized controlled trials (RCT) support the partial protective effect of male circumcision (MC) in reducing male HIV acquisition from an HIV-infected female sex partner by 53–75% [1–6]. The World Health Organization (WHO) and the Joint United Nations Program on HIV/AIDS promote MC as a key HIV prevention strategy where HIV prevalence and incidence are high and MC prevalence is low [7]. MC coverage of 80% of men 15–49 years is recommended to achieve the highest impact on HIV incidence [8].

In Zimbabwe, approximately 10% of the male population is circumcised [9]. In 2009, the country initiated a national MC program based on the forceps-guided surgical procedure, which resulted in approximately 50,000 circumcisions through the program’s end in 2011. However, the Ministry of Health and Child Care (MOHCC) estimates that 1.26 million circumcisions should still be performed by 2015 to achieve optimal MC coverage [10]. Alternative solutions to accelerate MC scale-up were sought. In October 2011, MOHCC commissioned a 3-phase RCT of the PrePex^TM^ device (*Circ MedTech Limited*) in order to determine its safety, performance, and ease of use (the device involves a plastic ring with a rubber O-ring that necrotizes the foreskin to facilitate easy and minimally invasive removal). The trial included: 1) a safety phase to determine the safety and efficacy of the PrePex device; 2) a comparative phase to assess the performance of the PrePex method compared to surgical circumcision; and 3) a field study to assess the ease of use by trained nurses. The safety phase results demonstrated that the PrePex device is safe for MC in Zimbabwe [11]. The current article presents the Phase II comparative study that assessed the PrePex device performance (safety, procedure time, and cost) compared to surgical circumcision.

## Materials and Methods

### Study design

This phase II, randomized, open-label trial compared the performance of the PrePex device to forceps-guided surgical circumcision in healthy adults using pre and post-circumcision measures over a 9-week study period (S1 Trial Protocol; S1 CONSORT Checklist). A randomized design was chosen for phase II to avoid multiple sources of bias arising from the inability to separate trial effects (such as patient selection, trial eligibility, and assessment schedule, and treatment locations) from treatment effect on clinical outcomes [12].

Power and sample size calculations were based on the primary endpoint of total procedure time. As we intended to compare two independent group means for procedure duration, we used the following formula to derive the sample size in each group: N = {4 (σ)^2^ [Z_crit_ + Z_pwr_]^2^} / D^2^ where σ is the assumed SD of each group, the Z_crit_ value is the desired significance criterion (0.05), the Z_pwr_ value is the desired statistical power (80%), and D is the minimum expected difference between the two means: SD = 15; Z_crit_ = 1.645; Z_pwr_ = 1.96; and D = 20–8 = 12. Thus N = {4 (15)^2^ [1.645 + 1.96]^2^} / 12^2^ = 81.2, which was rounded up to 80 for the surgical arm and 160 for the device arm for a 1:2 ratio. An unbalanced randomization of 2:1 was used to accumulate more data on the new PrePex device and is also in keeping with the WHO framework for clinical evaluation of devices for male circumcision.

Participants were randomly assigned by a simple randomization method to either the PrePex (probability = 2/3) or surgical arm (probability = 1/3). Safety, pain, compliance, satisfaction with the procedure and cosmetic results, time to complete healing, and cost-effectiveness were secondary outcomes measured over the 90-day study.

The study included clinical, psychosocial, and costing components. The clinical component involved trained doctors using the PrePex device on participants, who were observed and interviewed over multiple review visits to determine the safety and efficacy of the device. The psychosocial component involved private participant interviews by trained psychosocial interviewers to examine psychosocial factors associated with the procedures. Survey measures were assessed immediately before circumcision, 14-day post-circumcision, and 90-day post-circumcision to document and track changes in attitudes towards and satisfaction with circumcision.

The costs of the procedures were compared under the costing component. For the surgical arm, 3 routine follow-up visits were assumed; for the device arm, 4 were assumed. Unit cost data for commodities and consumables were collected from the study procurement records, the Supply Chain Management Services commodities forecast, and Population Services International. In some instances, unit cost was based on an average price for the item. Costs were compared for cost-effectiveness with a cost-analysis endpoint related to training, personnel time, infrastructure, tools, and materials. Indirect operational costs and overhead costs were excluded, but have been assumed to be the same for both arms, and so, would not impact the findings.

### Study site

The MC study site was Spilhaus Centre, a family planning clinic in Harare, which provides free surgical MC. The reception area, the examination area, the operating room, and the procedure room are dedicated to performing 15–20 MCs daily, but can perform as many as 40–60 daily MCs. Counseling rooms and waiting areas are shared with family planning clients. Nurse employees were trained for study participation and provided an extra allowance. Doctors were employed on a locum basis and paid on a daily basis.

### Study population

Male adult residents in Harare aged at least 18 years, who were scheduled for voluntary circumcision, were targeted for enrollment. Only participants who signed the Medical Research Council of Zimbabwe’s (MRCZ) approved Informed Consent Form (ICF) and met all eligibility criteria qualified for trial enrollment.

The inclusion criteria were: Male aged ≥18 years; uncircumcised and agreement to be circumcised; HIV sero-negative; ability to understand study procedures and requirements, and freely give informed consent for participation in this study; agreement to abstain from sexual intercourse and directly rubbing circumcised area (up to 70 days post-procedure) and until the end of the follow-up, and to return to the healthcare facility for follow-up visits (or as instructed); agreement to follow instructions about sexual abstinence and return visits; and consideration by the investigator to have potential for good compliance for the duration of the study based on (1) type of employment—e.g., a cross border trader will often be traveling to neighboring countries and may miss review visits; and (2) place of residence; those of no fixed abode, those residing out of Harare and too far from Spilhaus clinic were likely to find it difficult to come for multiple review visits required for the study.

Participants ineligible for this study included those that met any of the following exclusion criteria: active genital infection, anatomic abnormality, or other condition, which in the opinion of the investigator prevented the participant from undergoing a circumcision; having phimosis, paraphimosis, warts under the prepuce, torn or tight frenulum, narrow prepuce, hypospadias, epispadias; known bleeding/coagulation abnormality; uncontrolled diabetes; did not agree to anonymous video and photographs of the procedure and follow-up visits; HIV sero-positive; refusal to take HIV test; and <18 years.

Reasons for each participant’s exclusion were recorded.

### Randomization

Upon trial enrollment, participants were randomly assigned to either the PrePex or surgical arm. Numbered marbles (1–240) were placed in a box. The data manager generated an Excel spreadsheet list of randomly assigned study arm numbers, which was given to the nurses. The participant picked one numbered marble from the box, which was then set aside. The participant was assigned that number. The nurse verified which study arm the participant was assigned to according to the randomization list. If a participant opted out of the assigned arm, he was excluded from the study. His marble was put back in the box.

### Training

PrePex Masters from Rwanda conducted the training using the most up-to-date program and certified 3 PrePex operators (physicians, including 1 urologist, 1 general surgeon, and 1 general practitioner) and 4 assistants (nurses). All 3 PrePex operators and two assistants (nurses) were actually trainers of trainers on the national surgical MC training program. They completed surgical training in 2009–2010, were all trained on PrePex during the phase I trial and then retrained for the phase II trials. The nurses were senior-registered general nurses with over 10 years of experience. The training program included didactic and practical sessions to ensure that each operator successfully participated in at least 10 device applications and 10 device removals. All operators underwent one-day protocol training to familiarize themselves with the ICF, Case Report Forms (CRF), and other data collection tools. This was an additional day for practical sessions on the protocol. Before this day the team had a 1-week protocol and ethics training conducted jointly by the research council of Zimbabwe, the external data monitoring team from Malawi and the project principal investigators. The PrePex training was done separately and this took about 2 weeks covering theory and practical application.

The national MC program runs regular surgical circumcision training sessions, which were used in this trial. The program included 2.5 days of MC theory and 3.5 days of practical sessions to ensure that each doctor successfully performed at least 12 circumcisions using the recommended forceps-guided method.

### PrePex device and procedure

The PrePex device is composed of an inner ring, elastic ring, placement ring and a sizing accessory. All components are single-use (disposable). Circ MedTech Limited supplied the PrePex devices, sizing plates, spatula, scissors, forceps, and Lidocaine cream. All other materials were procured from local pharmaceutical and medical suppliers in Zimbabwe.

The procedure was performed in a clean, non-sterile environment in 2 stages: device application and foreskin/device removal, which followed the same protocol of an earlier RCT [13], except that dermal anesthetic cream (5% Lidocaine) was applied during placement to avert 1-hour post-placement discomfort. After device placement, a packet of 18 tablets of Paracetamol was given to each participant to take as needed (dosage: 2 tablets, 3 times a day).

After the foreskin and device removal, participants were instructed not to touch the dressing and to return for a follow-up visit 2 days post-removal for dressing removal and documentation of any adverse event (AE) and/or pain. Paracetamol was given to participants who continued to feel pain. Participants underwent weekly follow-up visits for routine documentation of AEs, pain, and healing status of circumcision site until complete healing was achieved. Complete healing was defined as complete epithelialization with no drainage from the circumcision site as per WHO Framework for Clinical Evaluation of Services for MC.

### Forceps-guided surgical circumcision procedure

All surgical circumcisions were performed under local anesthesia using the forceps-guided method consistent with the national guidelines adapted from the WHO Manual for Male Circumcision under Local Anesthesia Version 2.5C January 2008 [14].

### Follow-up visits

For the PrePex arm, a standard visit schedule was used that included a screening and device application visit on day1, foreskin and device removal visit on day 7, and weekly follow-up visits thereafter until wound healing (Table 1). An additional visit was scheduled on Day 90 for psychosocial interviews. For the surgical arm, follow-up visits took place on days 3, 7, and 42, which are the established “3 times” review schedule for clients undergoing surgical circumcisions under the national VMMC guidelines (Table 1). Participants in this arm were asked to make two additional visits on day 14 and day 90 for psychosocial interviews. Participants in the surgical arm were instructed to use salt sitz baths after the removal of the dressing on day 2 post-surgery until wound healing was achieved.

**Table 1:** Description of visits for participants in both surgical and PrePex device arms.

	Screening & Random-ization	Device Application or Surgery	Physical Exam of Genitals	Device Removal	Physical Exam of Genitals	Physical Exam of Genitals	Physical Exam of Genitals	Physical Exam of Genitals	Physical Exam of Genitals	Final visit
Day	–7 to 0	0	3	7	9	14	21	28	35, 42, 49, 56, 63	90
Informed consent	**X/O**									
Screening	**X/O**									
Medical history & Demography	**X/O**									
Current medication, including pain killers	**X/O**	**X/O**	**X/O**	**X/O**	**X**	**X**	**X**	**X**	**X/O**(D42 only)	
Psychosocial interview	**X/O**					**X/O**				**X/O**
Genital exam and pictures	**X/O**	**X/O**	**X/O**	**X/O**	**X**	**X**	**X**	**X**	**X/O**(D42 only)	
Video procedure		**X**		**X**						
Device application		**X**								
Foreskin removal				**X**						
Device removal				**X**						
Pain and discomfort evaluation		**X/O**	**X/O**	**X/O**	**X**	**X**	**X**	**X**	**X/O**(D42 only)	
Expected side effects and AEs		**X/O**	**X/O**	**X/O**	**X**	**X**	**X**	**X**	**X/O**(D42 only)	
Analysis of data										Post 90-days

X: Device group procedure and follow-up visits; O: Surgical group procedure and follow-up visits

All participants were asked during each visit if they experienced any AEs and/or pain. Psychosocial interviews of participants occurred during screening, and on days 14 and 90.

### Data collection

Data were collected from all participants using standard CRFs (case report forms) and interviewer-administered questionnaires. The 2 arms were compared in regards to the following parameters: Procedure operative time (device placement through device removal for the PrePex arm) or first cut to last suture for the surgical arm, including administration of anaesthesia; preparation time; pain and or discomfort at key time points pre-, during, and post-procedure assessed by the Visual Analogue Scale (VAS); clinical AE rates, defined as: site bleeding, penis-diffused hematoma, penis-diffused edema, incision site infection; device-related AE rates defined as: necrotic process did not initiate, device did not remain in situ for the full 7 days (mild events considered common to any MC procedure were not counted as AEs, whereas the AE totals include events graded as moderate or severe events in keeping with the PrePex MC Classification of Adverse Events and Device Hazards, Version 01, Dated Jan 18 2012); participant and partner satisfaction (the healing process, no complaint of pain, husband/partner involvement in routine activities of daily living during the healing period) based on a 5-point scale ranging from ‘not at all satisfied’ to ‘extremely satisfied’; time to complete healing based on treating physician’s objective wound status evaluation by scores of different wound parameters, such as exudate/tissue type; difficulties and complications during procedure based on operator reports; and cost of procedure based on cost-analyses related to training, personnel, time, infrastructure, tools, and materials.

### Data entry and processing

Data entry screens were designed in SPSS with logic checks and skip patterns. The Zimbabwe Community Health Interventions and Research (ZiCHIRe) office provided data management, record maintenance, data checking and cleaning, and double entry verification for all quantitative trial data. Data were backed up on the ZiCHIRe servers and external drives.

### Data analysis

The primary endpoint evaluations were performed according to intention to treat. Categorical variables were presented across both arms and within each arm as frequencies and percentages. Confidence intervals (95%) for the incidence of AEs were calculated by an exact binomial method. Continuous variables were summarized by mean, median, standard deviation (SD; 2-sided 95% confidence intervals [CI]), and range). Chi-square/Fisher exact tests (proportions) or t-tests (quantitative variables) were used to compare rates or means, as appropriate. The study was designed with a 0.05 alpha level. P-values of <0.05 were considered therefore significant. Simple descriptive analyses were conducted for all psychosocial variables of interest.

Key assumptions were made to calculate the unit cost for the surgical and device procedures. Shared costs were allocated to circumcision using allocation factors, such as share of staff time or square metres of facility space used. A cost minimization analysis was done using the Male Circumcision Costing Workbook (MC Workbook) [15] developed by Futures Institute. Capital and initial training costs were annualized. Data analysis was undertaken jointly with the research team, which verified each cost component, the model assumptions, and the final unit costs.

### Ethical conduct

This study was conducted in accordance with the Declaration of Helsinki [16]. Participants provided their written formal consent and confirmed their understanding of their participation. Medical Research Council of Zimbabwe’s (MRCZ) and Battelle’s Institutional Review Board approved this study (MRCZ: approval September 5, 2011, MRCZ/A/1628; Battelle: approval October 2011, IRB000284). All interviewers and nurses attended study protocol training sessions, as well as a Good Clinical Practices (GCP) course offered jointly by the Research Support Centre, College of Medicine of Malawi, and MRCZ. The study was monitored by international monitors (Research Support Centre, College of Medicine of Malawi) for Good Clinical Practices and international standards, such as ISO 14155. A Data Safety Monitoring Board was established to provide required oversight in all phases of the trial. The trial was registered at clinicaltrials.gov (NCT01956370); the delay in registering the study with clinicaltrials.gov, which occurred after enrolment started, was caused by the delay in obtaining the letter of support from the Zimbabwe Ministry of Health. The authors confirm that all ongoing and related trials for this drug/intervention are registered. The United Nations Population Fund in Harare funded this study and did not have any other role.

## Results and Discussion

**Fig 1:**
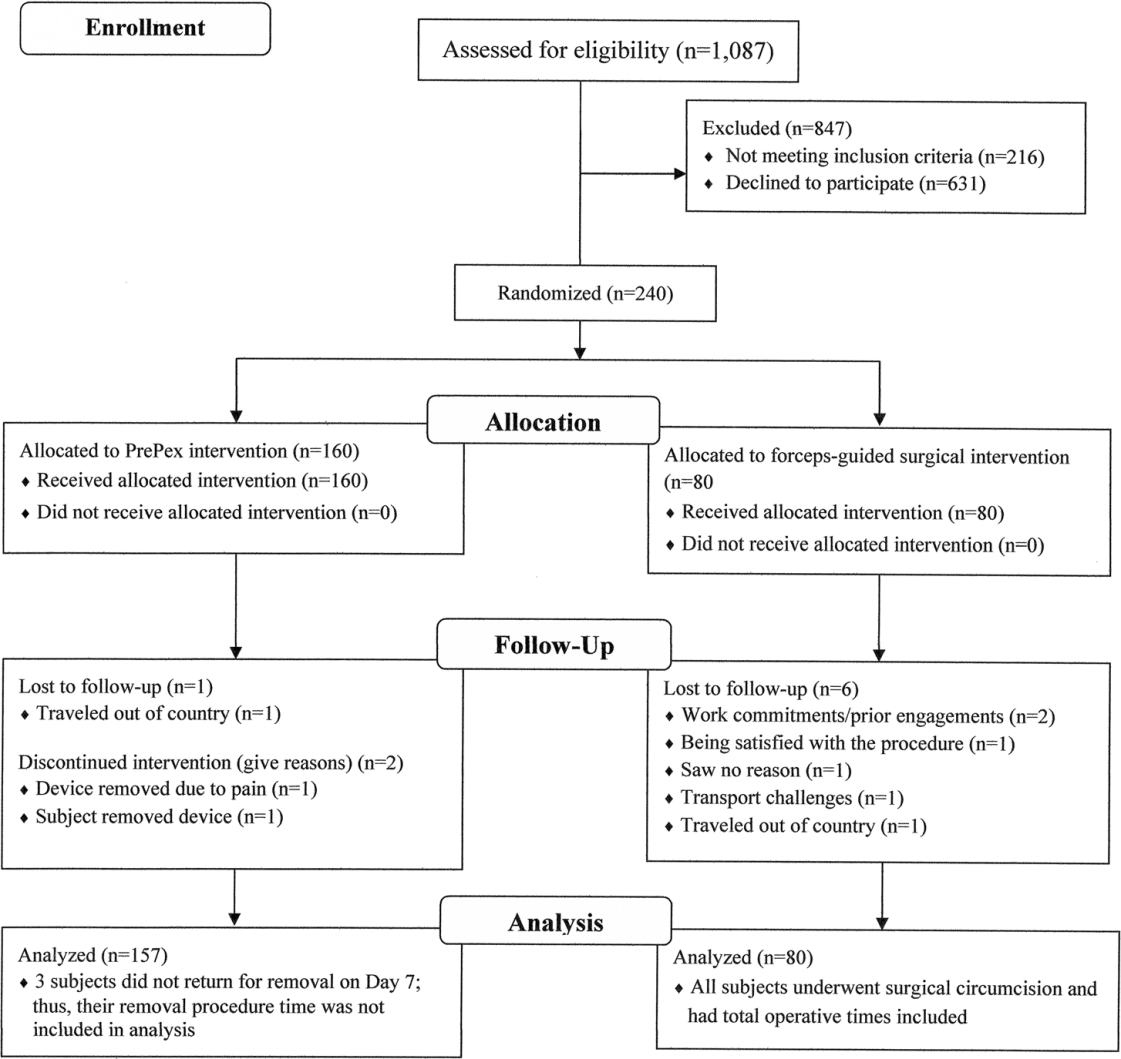
Flow chart summarizing the screening and enrollment of the male participant candidates.

Participants were recruited, enrolled, and underwent MC and follow-up over a period of 58 days from November 21, 2011 to January 18, 2012 (S1 CONSORT Checklist). There were 1,087 men screened for eligibility; 216 men were excluded for being <18 years, and 631 men declined to participate, including 12 who declined after being randomly allocated to the surgical arm (Fig 1). There were 240 participants randomized to the device arm (n = 160) or the surgical arm (n = 80), all of whom received the intervention allocated. All participants in the surgical arm (n = 80) and 98.1% in the device arm (n = 157) were included in the analysis. Three participants in the device arm did not return for device removal on day 7, and thus were not included in the analysis of the clinical and psychosocial components of the study (see AE section regarding the first 2 participants; participant 3 was lost to follow up as he travelled out of Zimbabwe to South Africa with the device still in situ and reportedly had it removed there on day 8 by a medical officer who obtained removal guidelines from the manufacturer’s website. He reported at Spilhaus on day 90 and was observed to be completely healed and was discharged.

### Sociodemographics

The mean age of participants was 29.1 years (SD: 9.0) for the PrePex arm and 27.6 years (SD: 7.6) for the surgical arm (Table 2). The difference in mean age between the 2 study arms was not significant (p = 0.198). Nearly half of participants were single (49.2%) and most were employed (informally: 24.2%; formally: 48.8%).

**Table 2:** Socio-demographic characteristics of participants in both surgical and PrePex device arms.

Characteristic	Assigned Study Arm Surgical arm, n = 80 (%)	Assigned Study Arm PrePex arm, n = 160 (%)	Total (%)
Age 18–29 years	52 (65.0)	111 (69.4)	163 (67.9)
Age 30–49 years	28 (35.0)	44 (27.5)	72 (30.0)
Age 50+ years	0 (0)	5 (3.2)	5 (1.9)
Mean ± SD^a^	27.6 ± 7.6	29.1 ± 9.0	
Single/never married	44 (55.0)	74 (48.1)	118 (49.2)
Married	34 (42.5)	79 (49.4)	113 (47.1)
Divorced	2 (2.5)	5 (3.1%)	7 (0.9)
Widowed	0	2 (1.3)	2 (0.8)
Unemployed	27 (33.7)	43 (26.9)	70 (29.2)
Employed	3 (66.3)	117 (73.1)	170 (70.8)
Formal employment	35 (43.8)	73 (45.6)	108 (63.2)
Informal employment	18 (22.5)	44 (27.5)	62 (36.5)
Harare, high density	47 (58.5)	70 (43.8)	117 (48.8)
Harare, medium density	3 (3.8)	25 (15.6)	28 (11.7)
Harare, low density	15 (18.8)	43 (26.9)	58 (24.2)
Harare town center	1 (1.3)	3 (1.9)	4 (1.7)
Out of Harare, medium density	14 (17.5)	19 (11.9)	33 (13.8)

^a^SD: standard deviation.

### Size of PrePex device

A total of 160 devices were successfully applied. The PrePex device is available in 5 sizes: A (2.6 cm), B (2.8 cm), C (3.0 cm), D (3.2 cm), and E (3.4 cm). The most common sizes used were C (67 participants; 41.9%) and B (52 participants; 32.5%) followed by A (14; 8.8%), D (25, 15.6%), and E (2; 1.3%).

### Total preparation and procedure times for both arms

PrePex preparation time included the time to take off pants, drape (optional), disinfect (both visits), and dress the wound. The surgical arm preparation time included draping (optional), disinfecting, anesthesia, and wound dressing.

The overall procedure time included application and removal times for the PrePex procedure and the time from first cut to last suture for the surgical procedure, as well as preparation times for both procedures (Table 3). The mean total procedure time for the PrePex device was approximately one-third of the total surgical procedure (4.8 minutes, SD: 1.2 versus 14.6 minutes, SD: 4.2; p<0.00001).

**Table 3:** Total procedure time (in minutes) for participants in surgical and PrePex device arms.

Placement and Removal (Procedure)	Time (in minutes) Surgical arm (n = 80)	Time (in minutes) PrePex arm (n = 157)
Preparation, median (Q1, Q3)	6.4 (5.0, 8.0)	1.9 (1.6, 2.3)
Preparation, mean ± SD^a^	6.6 ± 1.8	1.9 ± 0.4
Procedure, median (Q1, Q3)	6.0 (5.0, 8.0)	2.6 (2.3, 2.5)
Procedure, mean ± SD^a^	6.6 ± 2.2	2.9 ± (1.0)
Total time, median (Q1, Q3)	13.9 (11.6, 17.6)	4.5 (4.0, 5.7)
Total time, mean ± SD^a^	14.6 (4.2)	4.8 ± 1.2
Placement, preparation, median (Q1, Q3)	Not applicable	1.6 (1.3, 2.0)
Placement, preparation, mean ± SD^a^	Not applicable	1.7 ± 0.4
Placement, median (Q1, Q3)	Not applicable	0.9 (0.7, 1.2)
Placement, mean ± SD^a^	Not applicable	1.0 ± 0.4)
Placement, total, median (Q1, Q3)	Not applicable	2.6 (2.1, 3.2)
Placement, total, mean ± SD^a^	Not applicable	2.7 ± (0.7)
Removal, preparation, median (Q1, Q3)	Not applicable	0.3 (0.2, 0.3)
Removal, preparation, mean ± SD^a^	Not applicable	0.3 ± 0.2
Removal, median (Q1, Q3)	Not applicable	1.7 (1.4, 2.2)
Removal, mean ± SD^a^	Not applicable	1.9 ± 0.8
Removal, total, median (Q1, Q3)	Not applicable	2.0 (1.6, 2.5)
Removal, total, mean ± SD^a^	Not applicable	2.2 ± 0.8

^a^SD: standard deviation.

### AEs

The AE incidence rate for the PrePex arm was 1.3% (95% CI: 0.0025–4.53%), comprising 2 AEs in all participants that were mild, device related, and which occurred between days 0 and 7; all were quickly resolved. There were no AEs in the surgical MC arm and severe AEs in either arm.

AE#1: Participant returned to the site on Day 2, complaining of device-related pain. The device was removed. The participant underwent surgical circumcision. He healed successfully.

AE#2: Participant removed the device at home on the same day of application before foreskin necrosis occurred. He returned to the site on Day 3 and requested device reapplication, which was denied. The study urologist observed that the penis was normal without edema or necrosis. Surgical circumcision was offered and declined.

### Expected side effects

On Day 7, 19 (12.1%) participants in the PrePex arm presented with localized edema during device removal. On day 3, 5 (6.3%) participants in the surgical arm had localized edema, and 1 (1.3%) presented with mild wound oozing.

### Time to complete healing

A total of 237 participants were safely circumcised by the PrePex (n = 157) and by the forceps-guided surgical (n = 80) methods. By day 42, 87.3% of participants had healed in the PrePex arm compared to 76.3% in the surgical arm. The difference between the proportions healed was significant (p = 0.048).All participants had healed by Day 49 for the PrePex arm and Day 56 for the surgical arm.

### Pain assessment

During the procedures, all 160 participants in the PrePex arm reported no pain (VAS score = 0). All 80 participants in the surgical arm experienced some pain during the injection of local anesthetic, with over 90% reporting a VAS score between 4 and 6 (Table 4).

**Table 4:** Summary of VAS^a^ pain level at various points in time. Number and percentages of participants experiencing a given pain level (0, 2, 4, 6, 8).

Time of Occurrence	N	0 (%)	2 (%)	4 (%)	6 (%)	8 (%)	Missed Visits (%)
During procedure, PrePex arm	160	160 (100)	0	0	0	0	0
During procedure, Surgical arm	80	0	7 (8.8)	49 (61.3)	24 (30.0)	0	0
Post-procedure (15 min), PrePex arm	160	160	0	0	0	0	0
Post-procedure (15 min), Surgical arm	80	76 (95.0)	2 (2.5)	1 (1.3)	1 (1.3)	0	0
Post-procedure (1 hour), PrePex arm	160	160 (100)	0	0	0	0	0
Post-procedure (1 hour), surgical arm	80	78 (97.5)	1 (1.3)	1 (1.3)	0	0	0
Post-procedure (2 hours), PrePex arm	160	160	0	0	0	0	0
Post-procedure (2 hours), surgical arm	80	78 (97.5)	1 (1.3)	0	1 (1.3)	0	0
Post-procedure (3 hours), PrePex arm	160	160	0	0	0	0	0
Post-procedure (3 hours), surgical arm	80	69 (86.3)	7 (8.8)	3 (3.8)	0	1 (1.3)	0
Post-procedure (2 days), PrePex arm	157	116 (73.8)	39 (24.8)	1 (0.6)	1 (0.6)	0	3 (1.9)
Post-procedure (2 days), surgical arm	80	36 (45.0)	30 (37.5)	10 (12.5)	3 (3.8)	1 (1.3)	0
During erection (Day 3), PrePex arm	157	76 (48.4)	74 (47.1)	6 (3.8)	1 (0.6)	0	3 (1.9)
During erection (Day 3), surgical arm	80	23 (28.8)	41 (51.3)	15 (18.8)	1 (1.3)	0	0
During erection (Day 3), p-value		0.0038	0.5407	0.0001	0.5758		
Post-procedure (7 days), PrePex arm	157	46 (29.3)	109 (69.4)	1 (0.6)	1 (0.6)	0	3 (1.9)
Post-procedure (7 days), surgical arm	74	60 (81.1)	12 (16.2)	1 (1.4)	1 (1.4)	0	6 (7.5)
Post-procedure (42 days), PrePex arm	44	44 (100)	0	0	0	0	19 (11.9)
Post-procedure (42 days), surgical arm	61	61 (100)	0	0	0	0	18 (22.5)

^a^VAS: Visual Analogue Scale.

Nearly all PrePex participants (93.6%) reported transient pain (VAS score: 2–6) during device removal; 66.5% reported pain after removal. On day 14 follow-up visit, 1.3% of participants in the PrePex arm still expressed level 2 pain score (little pain) compared to 16.2% of participants in the surgical arm.

Sixty-two participants (39.5%) felt the pain during erection in the PrePex arm while in the surgical arm, 44 participants (55%) felt pain during erection.

### Satisfaction

In the two post-procedure interviews (2 weeks and 90 days), men in the surgical and device groups were asked to rate their satisfaction regarding their circumcision on 5-point scales ranging from “not at all satisfied” to “extremely satisfied.” When 30 men in the surgical group were asked about their satisfaction with their circumcision, in the 2-week post-procedure interview, approximately 78% of men (23 out of 30) were “very” or “extremely” satisfied, with the remaining 7 (23%) being “less than very satisfied” compared to 70% of men (74 out of 104) in the device group who were “very” or “extremely” satisfied, with almost one third less so. This difference in satisfaction is not statistically significant (p > .05)

When men in the surgical group were asked about satisfaction with their circumcision, in the 90-day post-procedure interview almost all men (50 out of 51) indicated that they were satisfied with the circumcision, with about 94% (48 out of 51) indicating that they were “very” or “extremely” satisfied. Similarly, 99% of men from the Prepex arm (109 out of 110) indicated that they were satisfied with the circumcision, with about 88% indicating that they were “very” or “extremely” satisfied. This difference in satisfaction between the two groups is not statistically significant (p > .05)

### Costs

Unit cost components (in USD) are compared for the PrePex and surgical circumcisions in Table 5. The estimated total costs were $45.99 and $54.26 per PrePex and surgical circumcision respectively and without complications. Consumables and direct staff costs contributed to more than 90% of the total costs. The device is the most costly of PrePex commodities at $18.00 (including procurement and distribution costs). For surgical circumcisions, the kit is the main commodities costs at $19.56.

**Table 5:** Male circumcision unit cost components for PrePex device and surgical circumcisions for Phase II.

Item	PrePex Circumcision (USD)	% of Total Circumcision Cost	Surgical Circumcision (USD)	% of Total Circumcision Cost
Consumable supplies	27.92	60.8%	29.66	54.7%
Non-consumable supplies	0.41	0.9%	0.37	0.7%
Personnel	16.38	35.7%	22.69	41.8%
Support personnel	0.80	1.7%	0.80	1.5%
Training	0.18	0.2%	0.27	0.5%
Capital	0.30	0.7%	0.48	0.9%
Total unit cost	45.99	100%	54.26	100%

### Discussion

The trial results demonstrate that the PrePex procedure is quick, efficient, and effective at approximately one-third the time of the surgical procedure. There were no severe AEs, although there were 2 device-related AEs which were due to participants removing the device (1.3%). These AEs were successfully resolved without any complications, further demonstrating the safety of PrePex device, even when removed by inquisitive users. In the largest study conducted to date in Uganda, this type of event occurred in 5 out of 625 participants (0.8%), which is considerably lower [17]. The device-related AE (1.3%) was higher than the rates reported in Rwanda, which ranged from 0% to 0.77% [12,18,19], but lower than device-related AE rates reported in the large observational studies from Uganda and Kenya (2.3% and 6.8%. respectively) [20,21]. The clinical effectiveness of the device in this study as defined by WHO would be 98.7%, which emphasizes the point that surgical circumcision services will still be necessary unless effectiveness is 100%. However, we note that the sample size of this study, as well as the others published to date, does not allow for very precise estimation of AEs, making comparisons of AE rates difficult and the estimation of clinical effectiveness imprecise.

The PrePex procedure time required approximately one-third the time of the surgical procedure (4.8 minutes versus 14.6 minutes), which is consistent with results reported in Rwanda [14,15], Uganda [20] and Kenya [21]. Participants in the surgical arm experienced more pain than those in the PrePex arm. Less pain during erection in the PrePex arm may be attributed to the fact that the PrePex Inner Ring diameter is designed to be larger than the measured penis sulcus.

Six weeks after surgery or device application, 87% of PrePex circumcisions healed compared to 76% of surgical circumcisions, which is a little longer than the expected range of the healing time of 4–6 weeks after surgical circumcision in adult males [14]. Our trial results are also different to those obtained from the randomized controlled trial conducted in Rwanda in which healing was significantly faster in the surgical arm compared to the device arm [18]. Although surgical time and AE rates are similar between the 2 trials, in Rwanda, the surgeries were performed by a highly experienced surgeon who used the dorsal slit method rather than the forceps guided method. While the forceps-guided method is faster than the dorsal slit method and requires less skill, the latter method is likely to lead to less trauma, which could have resulted in faster healing.

Overall satisfaction with the circumcision was high at the 2-week interview and increased by 90 days, with a trend of greater increase in satisfaction in the surgery arm

The unit cost of a PrePex procedure was $8.27 less than that of surgical circumcision. These findings are similar to those reported in Rwanda [18] and have important implications for rapid MC scale-up in Zimbabwe and other resource-limited settings, as PrePex circumcision can be more cost-effective than surgical circumcision. In Kenya, cost-effectiveness of the PrePex device was dependent on a number of issues, including the proportion of participants with phimosis or tight foreskins in which dorsal slit circumcision surgery would be necessary, the cost of the device and the number of ring sizes [21,22]. To be fully cost-effective, staff utilization must be also maximized and sites need to function at maximum capacity to take advantage of improvements in operator efficiency, thus achieving the best cost-effectiveness [23,24]. In Zimbabwe, PrePex circumcision could potentially be done more by nurses than doctors, therefore becoming more accessible at peripheral health facilities and less costly [25].

The study limitations include the inadequate follow-up times for the surgical arm participants to allow for accurate assessment of the healing time, and the low participation response on satisfaction post-procedure (surgical arm: 37.5% at 2 weeks post-procedure and 63.8% at 90 days post-procedure; PrePex arm: 65% at 2 weeks post-procedure and 68.8% at 90 days post-procedure). For the cost analysis, personnel costs included were not calculated at the official government salary scale, but they approximate those used by the existing program and the rates paid during the study. If the program is scaled up based on government pay scales and no allowances are paid, the staff costs would be much lower. It is unlikely, however, that the government pay scales are sustainable over the life cycle of MC scale-up due to being very low. AE costs were excluded from the costing analysis, as insufficient data were collected to accurately calculate them. The time taken to resolve AEs was not accurately recorded.

### Conclusions

The trial results support previous studies’ conclusions that the PrePex procedure is safe, quick and easy to apply, effective in terms of surgical time, and cost-effective as an alternative to surgical circumcision. The PrePex device has great potential for use in overburdened health systems, in resource-limited settings, and for adult MC scale-up in Zimbabwe. However, future studies will be needed to define efficacy in regards to prevention of HIV transmission.

## Supporting Information

S1 CONSORT Checklist CONSORT checklist for the trial with match of questions to manuscript sections. (DOC)

S1 Trial Protocol The approved trial protocol. (PDF)
