# Supplementary material for: A Phase II Randomized Controlled Trial Comparing Safety, Procedure Time, and Cost of the PrePex™ Device to Forceps Guided Surgical Circumcision in Zimbabwe
Source: PLoS One. 2016 May 26;11(5):e0156220. doi: 10.1371/journal.pone.0156220 (PMC4881993; doi:10.1371/journal.pone.0156220)
Supplement: S1 Trial Protocol — (PDF) [file pone.0156220.s002.pdf]

Protocol Title

Evaluation of Safety and Efficacy of the PrePex^{TM} Device for Rapid Scale up of Adult Male Circumcision Programs in Zimbabwe

Protocol Date

June 19, 2011

Protocol Version

Version 04

Participating Investigators

1. Dr Gerald Gwinji**,** MBChB, MPH. PS- MoHCW, **Principal Investigator**
2. Prof Mufuta Tshimanga, MD; Public Health Physician, Department of Community Medicine, University of Zimbabwe. **Co-Principal Investigator**
3. Dr. Mangwiro,MMed Urology, Department of Surgery, University of Zimbabwe
4. Dr Owen Mugurungi, MBChB, Msc; Director AIDS- TB , MoHCW
5. Dr Munyaradzi Murwira, MBChB, MPH. Director ZNFPC
6. Dr. Danuta Kasprzyk,PhD, Research Leader, Battelle
7. Dr. Daniel Montaño, PhD, Research Leader, Battelle
8. Mrs Daisy Nyamukapa, Program Officer, UNFPA
9. Mr Sinokuthemba Xaba, MC Program Officer, AIDS-TB, MoHCW

A. Background of the study

A Global HIV Prevention Working Group (GHPWG) estimated that by fully scaling up all scientifically proven HIV prevention strategies, approximately 30 million of the 60 million expected infections to occur by 2015 could be averted. Adding MC to prevention programs would account for 8% of the drop in infections by 2015. This underscores the potential power of MC as a prevention tool, provided it is used in combination with other proven prevention strategies; including condoms, HIV testing, and PMTCT (GHPWG, 2007). Models show that to have the highest impact on the HIV epidemic, circumcision of up to 80% of men in high prevalence countries, where MC rates are low, needs to be achieved (UNAIDS 2009; Manicaland HIV/STD Prevention Project, 2008; Bollinger, et al, 2009). Hallett and colleagues in 2008 showed that for Zimbabwe specifically, HIV incidence could be reduced between 25% to 35% if about 50% of men are circumcised. All modelings conducted show that MC programs must be part of a comprehensive HIV prevention package as MC alone will not tip the HIV epidemic into a terminal decline (Hallett, et al., 2008; Bollinger, et al., 2009).

Zimbabwe is now implementing a national MC Programme after successfully launching five demonstration sites (Mutare, Karanda, Harare, Bulawayo, and Manyame) as a ‘proof of concept’, opening an MC Training Centre, and conducting about 11, 174 circumcisions during 2010 (MOHCW, 2010). MC has been integrated into Zimbabwe’s comprehensive prevention program. Current population-based estimates of MC in Zimbabwe show by self-report that about 10% of men are circumcised, with regional variation (CSO, 2007). MOHCW estimates that scaling up to reach 80% of men in Zimbabwe means that they must conduct over 1.2 million MCs in 2012, which will be the peak year for MC (Mugurungi, 2010).

Many countries are proactively seeking innovative solutions for scaling up male circumcision. The surgical method is inherently not scalable and to achieve the aforementioned target at the current yield would take several decades and intended prevention benefits may not accrue. The PrePex™ Device offers hope for rapid scale up of adult male circumcision in resource-limited settings. The Device uses a plastic ring with a rubber O-ring to necrotize the foreskin so it drops off. A Phase II safety trial was done in Rwanda (Bitega, et al, 2011) showing that the PrePex™ device is safe to use. Additional safety data is required to ensure that the device is safe to use, as well as acceptable to both clinicians and patients. Once the safety and efficacy have been established, it is important to conduct operations research to determine how best to implement both device and surgical circumcisions in the national program. Thus, the Zimbabwe MOHCW is planning a three phase trial in order to determine the safety of the PrePex™ device, its performance and the ease of use in an implementation roll out.

B. Objectives

Primary Objective for Phase I:

To assess the safety of the PrePex™ device by means of the following parameters: Clinical adverse events and device-related incidents.

Secondary Objectives for Phase I:
To evaluate the effectiveness of the PrePex™ device by means of the following parameters:

- Procedure duration,
- Pain at key time points,
- Discomfort during daily activities,
- Compliance during use (with follow-up),
- Procedure acceptability by Doctors,
- Procedure acceptability by Nurses,
- Satisfaction by staff,
- Satisfaction by subjects,
- Glans fully exposed,
- Time to complete healing,
- The cosmetic results,
- Performance targets.

Primary Objective for Stage II: To compare the total operative time of the PrePex Device circumcision procedure versus the total operative time of surgical circumcision procedure.

Secondary Objectives for Stage II:
To compare the surgical and PrePex™ non-surgical device circumcisions on the following parameters:

- Expectations of men regarding the procedure
- Satisfaction with procedure
- Attitudes towards procedure
- Sharing information with family and friends
- Perception of norms
- Sexual relations, performance, satisfaction
- Sexual behavior
- Sexual partner's attitudes
- Effect on daily activities
- Time off work
- Costs
- Cost-effectiveness

Primary Objective for Stage III:
To conduct a field trial of PrePex™ device implementation using trained nurses

Secondary Objectives for Stage III:

- Evaluate the training needs of PrePex™ deployment using nurses
- Observation of in-field usability of device
- Obtain user feedback from nurses
- Assess safety compared to physician deployment
- Assess practicality and acceptability among nurses and patients

- Assess satisfaction of procedure among nurses and patients
- Track costs and time of PrePex™ procedure
- Determine cost-effectiveness compared to surgical procedure

This protocol covers designs for stages I and II of the trial. A separate protocol for stage III will be submitted once safety of PrePex device is established.

C. Design of the Study

The evaluation of the PrePex™ device will be conducted in a three phase trial each of which has specific goals: 1) safety trial of the PrePex™: Device: The enrollment will be phased with completion of the initial small cohort of 5 men being systematically observed from device deployment to device removal for safety. Once safety of the device is established, additional cohort recruitment and phase 2 clinical trial of the device on 50 men will be done to further document clinical adverse events and device-related incidents. In addition to the safety and the performance of the device, other important considerations are the attitudes towards and acceptability of the device (during the procedure itself, during healing period while the device remains *in situ*, and regarding cosmetic finish) for the patient, his female partner, as well as the provider. 2) Comparative study, a randomized, open label, trial comparing the performance of PrePex™ system to surgical circumcision in healthy adults. This phase will also examine the psychosocial factors associated with each type of circumcision to determine men's expectations, their satisfaction with each procedure, their post-circumcision attitudes and the impact on their sexual behavior post procedure. In addition, inconvenience and time taken off work or interruption of normal daily activities will also be assessed. Costs of both procedures will be tracked for cost-effectiveness comparisons. 3) Field Study: a non-comparative field trial of the device on 500 participants or more, with procedures performed by trained nurses to evaluate the training needed to learn the device procedure, the usability of the device in a clinic setting, the cost-effectiveness of the device compared with the standard surgical technique, the safety of the device when used by non-physicians, as well as the practicality, acceptability and satisfaction with the procedure and device.

1. **Study Design for Safety Trial**
   The safety trial will be implemented at Parirenyatwa group of hospitals among 50 volunteer men who will be examined to ensure they are eligible for the device circumcision. Men will be observed and interviewed over multiple time periods. See Table 1 for study design and data collection time points. The primary safety endpoint is the incidence of Clinical adverse events and Device-related incidents.

2. **Study Design for Efficacy Trial:**
   Phase two will consist of a randomized, open label; trial comparing the performance of PrePex™ system to forceps guided surgical circumcision in healthy adults using pre- and post-circumcision measures. The subjects will be randomly allocated to two unbalanced study arms, the PrePex™ arm which will include about a hundred (160) subjects and the surgical circumcision arm which will include about fifty (80) subjects. This study will

provide comparison data on the total operative time (primary endpoint), pain, complications, as well as attitudes towards device and surgical circumcision, including acceptability for both procedures. Study duration per subject will be 9 weeks. The whole study is expected to be conducted in a period of 3 months. Men will be observed and interviewed over multiple time periods (See Table 1). The primary end-point is the overall procedure time.

3. Study populations

Male adult population aged between 18-30 years who are resident in Harare.

3.1. Source of Subjects and Enrollment

Male subjects scheduled for voluntary circumcision will be targeted for enrollment. Only subjects who have signed the Medical Research Council of Zimbabwe's approved Informed Consent Form (ICF) and meet all of the eligibility criteria listed below will qualify for enrollment. The study will recruit at least 5 evaluable subjects for the safety and efficacy study and 240 for the comparative study who will complete the protocol procedure /treatment and the follow-up schedule.

3.2. Inclusion Criteria:

Subjects eligible for this clinical study must fulfill all of the following:

- Ages 18 – 30 years
- Uncircumcised
- Wants to be circumcised
- Agrees to be circumcised by any of the study methods, PrePex™ or Surgical as appropriate
- HIV sero-negative
- Able to understand the study procedures and requirements
- Agrees to abstain from sexual intercourse and to keep caution not to directly rub the cut area if masturbating, for 8 weeks post removal (9 weeks total)
- Agrees to return to the health care facility for follow-up visits (or as instructed) after his circumcision for a period of 8 weeks post removal (9 weeks total)
- Subject able to comprehend and freely give informed consent for participation in this study and is considered by the investigator to have good compliance for the study
- Subject agrees to anonymous video and photographs of the procedure and follow up visits
- Agrees to stay overnight at the Hospital in order to follow pain measurements in the first 16 hours

3.3. Exclusion Criteria:

Subjects not eligible for this research study include those that have any of the following:

- Active genital infection, anatomic abnormality or other condition, which in the opinion of the investigator prevents the subject from undergoing a circumcision
- HIV sero-positive
- Subject with the following diseases/conditions: phimosis, paraphimosis, warts under the prepuce, torn or tight frenulum, narrow prepuce, hypospadias, epispadias
- Known bleeding / coagulation abnormality
- Uncontrolled diabetes
- Subject that to the opinion of the investigator is not a good candidate
- Subject does not agree to anonymous video and photographs of the procedure and follow up visits
- Refusal to take HIV test.

4. Sample size assumptions and estimates

4.1. Safety and Efficacy study

Primary Safety Endpoint- Unexpected Device Related Serious Adverse Events

With a sample size of 5 subjects if no Unexpected Device Related Serious Adverse Events occur in the study it will be possible to claim that the adverse event rate is not higher than 5.82% with 95% confidence (RE Carter and RF Woolson, Statistical design considerations for pilot studies transitioning therapies from the bench to the bedside, Journal of Translational Medicine 2004, 2:37).

Primary Efficacy Endpoint – Complete circumcision

The PrePex™ is efficient, if more than 85% of the participants will be circumcised by the method. This can be claimed with 95% confidence if at least 86 (86%) subjects achieve the success criterion (lower limit of the exact binomial 95% confidence interval)

The intention is to obtain at least 50 evaluable subjects.

4.2. Comparative study

Power and sample size calculations were performed based on the primary end-point of overall net procedure time, and in order to support the hypothesis that the PrePex™ performs comparable to or better than the forceps guided surgical procedure (Rwanda study report). We assumed the average procedure time of 8 minutes for the PrePex™ and 20 minutes for surgery.

It was then determined that a total of 82 patients are required to have a 95% chance of detecting, as significant at the 5% level (95% power) a

decrease in the primary outcome measure (overall net procedure time) from 20 in the surgical group to 8 minutes in the experimental group. To maintain allocation ratio of 2:1 between the PrePex™ and the surgery, the target sample size was set to 160 subjects for the PrePex™ procedure and 80 subjects for the surgical procedure

5. Enrolment of participants

The following represents the study schedule of events:

1. **Informed consent** – Will be sought from all potential candidates for enrollment to the study
2. **Assessment of eligibility** – Subjects will be evaluated for inclusion in the study according to inclusion and exclusion criteria. Subjects which do not meet the criteria will be offered circumcision out of the study procedures and records
3. **Circumcision** – by either PrePex™ or Surgical Method

5.1. Informed Consent Procedure

Men aged between 18-30 years who are referred for male circumcision by Start Centres and City Health clinics are eligible. A trained nurse or doctor will explain the procedure and follow up visits in detail including the need to take anonymous photographs and videos of the entire process from circumcision to complete healing. We will tell potential participants that the procedure will provide to them safe male circumcision and help the MoHCW to understand the best way to scale up male circumcision and make it more accessible as one of prevention strategies for HIV infection. Should the participant agree after being told the details of the procedure and follow up visits, the nurse will obtain informed consent and proceed with assessment for eligibility to Prepex method.

5.2. Assessment of eligibility

Potential participants will get screened through history taking for ill health including bleeding disorders, diabetes etc...Subjects will be evaluated for inclusion in the study according to inclusion and exclusion criteria. Genital examination will be done to exclude penile abnormalities and infections and check on the flexibility of the prepuce. Potential participants who do not meet the study criteria will be offered circumcision out of the study procedures and records

5.3. Intervention Allocation

For the comparative study, subjects will be randomly assigned by simple randomization method to either PrePex™ or Surgical arm. Using an unequal allocation of 2:1 of intervention to control, participants will be assigned to PrePex™ arm with probability $p=0.66$ and to surgical arm with probability $p=0.34$.

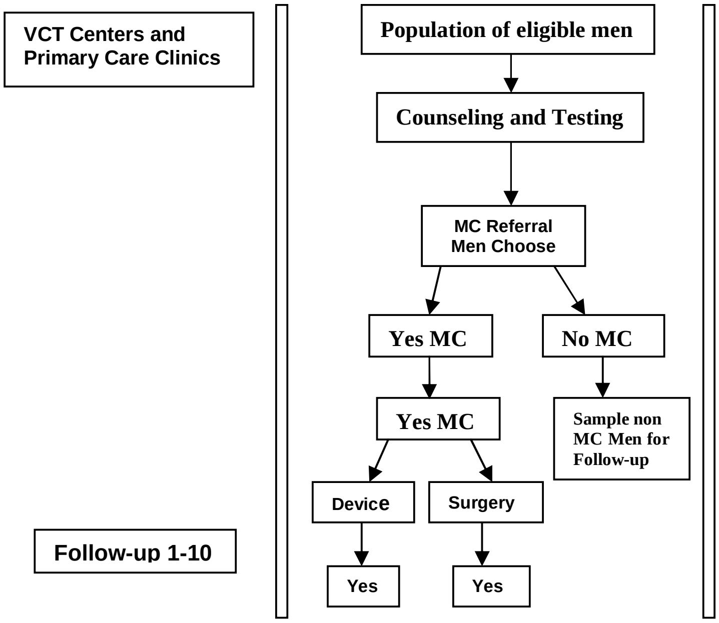

5.4. Subject Withdrawal Criteria

Subjects may be withdrawn from the study if any one or more of the following events occur:

- Subject wishes to withdraw from the study without providing any explanation
- Subject is lost to follow-up
- Refusal of the subject to continue treatment and/or follow-up observations
- Serious adverse event
- Significant protocol deviation/violation or noncompliance, either on the part of the subject or investigator
- A decision made by the investigator that termination is in the subject's best medical interest.
- Device failure or impossibility to continue surgical circumcision for any reason
- Other ethical or clinical considerations upon investigator discretion
- Significant protocol deviation/violation or noncompliance, either on the part of the subject or investigator

NOTE: The subject must know in advance that if he wishes to withdraw from the study, he must undergo surgical circumcision immediately upon removal of the device.

5.5. Handling of Withdrawals

In accordance with the current revision of the Declaration of Helsinki and local Authority regulations at the corresponding countries, a subject has the right to withdraw from the study at any time, for any

reason, without prejudice to his future medical care by the physician or the institution. The investigator and the DSMB also have the right to withdraw subjects from the study in the event of serious adverse events, protocol departure, or other reasons. Should a subject (or the subject's legally authorized representative) decide to withdraw, all efforts will be made to collect and report the final visit observations, and the reasons for withdrawal, as thoroughly and timely as possible. Withdrawals will be recorded, analyzed and reported to the local Ethical Committee (MRCZ).

6. Intervention

6.1. PrePex™ Circumcision Arm

6.1.1. Product Description

The PrePex™ device is intended to be used in a medical procedure to compress the foreskin of the penis to allow circumcision of an adult male. The device contains the following items:

(1) A Placement Ring (O-Ring Introducer) made of medical grade biocompatible plastic polymer that is highly used in the medical device industry.
(2) An inner ring made of medical grade biocompatible plastic polymer
(3) An (Elastic Ring) made of medical grade biocompatible elastic material.
(4) A verification thread inserted on the Elastic Ring

Devices will be supplied clean and disinfected. The use of the PrePex™ device should follow the 'Instruction for Use' supplied by CIRC MedTech and following an appropriate training to the operator and assistant.

6.1.2. Procedure and schedule

The procedure will be performed at Parirenyatwa Group of hospitals. Following the assessment of eligibility and after signing the informed consent form, the subject will be randomly allocated to one of the study treatment arms, PrePex™ circumcision or surgical circumcision for the comparative study.

- The subject will receive, 3gr of EMLA anaesthetic cream (J&J) before the procedure starts. The penile measurements will be taken in flaccid status. The penis will be cleaned by red Betadine solution (or other disinfectant solution), with the foreskin stretched backwards to expose the inner skin for the disinfection solution. Then the foreskin will be dried. The operator will then mark the circumcision line with a marker and then open the corresponding sized package of the PrePex™ System, and will load the Elastic-Ring on to the Placement Ring, The Placement Ring with the Elastic Ring will be placed on the penis shaft. Then, lubricant may be rubbed on the foreskin and glans and the foreskin tip will be stretched open to the sides by the operator hands with optional use of dry gauze to affirm the grip. Then, the inner ring will be applied through the stretched foreskin and over the glans penis (hereinafter, "glans") so that it will be placed fixed over the sulcus behind the glans (the base of the glans). At this point, the

foreskin around the inner ring is held tight, the Placement Ring and Elastic-ring is approximated to the inner ring. The foreskin is then adjusted to fit the circumcision marking line to the Elastic-Ring. Then, the Elastic-Ring is rolled from the Placement Ring on to the Inner Ring groove to fit in tight and press the foreskin. The procedure is then completed and the subject will be instructed to return at prescheduled follow up visits and in any non sustained situation. The subject will return 3 days following device placement for a follow up visit. The device will remain in place for 7 days, at day 7 the subject will return for a removal visit. At the removal visit, the foreskin is removed using clean scissors, the Elastic Ring is removed using a scalpel, and then the Inner Ring is extracted with hand or spatula. Following the removal process, the wound area will be dressed with a sterile gauze or non adherent pad for 2 days. Following the 2 days (day 9) the subject will return for a follow up visit and the dressing will be removed. The subject will return for a weekly visit (days 14, 21, 28, 35, 42, 49, 56 and 63) for 8 weeks. The subject will be instructed to return in any non sustained situation, regardless of the weekly follow-up visits scheduled.

6.2. Surgical circumcision arm (Comparison):

- The subject will undergo penile measurements at flaccid status. The penis will be surgically circumcised using the Forceps guided method consistent with the nationally adopted MC procedure. Where the forceps guided method is not appropriate the subject will be circumcised according to one of the WHO recommended circumcision methods as described in the Manual for Male Circumcision under Local Anaesthesia Version 2.5C January 2008. A standard penile dressing will be placed according to WHO guidelines. The surgeon who performs the circumcision will conduct the follow-up examination. The follow-up visit should be on days 2, 7 of surgery. After the removal of the dressing 48 hours post-surgery, the subject will be counseled/instructed to use sitz baths until wound healing is achieved. The investigator should assess the progress of healing and look for signs of infection. The operation site should be examined, and additional examinations should be done as required by the case history, symptoms or complaints of the subject. If complications occur during or after the circumcision, the team should take the time to inform the subject, and if possible his family, about what has happened and the plans to deal with the complication. The subject will return for prescheduled weekly visit (days 14, 21, 28, 35, 42, 49, 56 and 63) for 8 weeks. The subject will be instructed to return in any non sustained situation, regardless of the weekly follow-up visits.

7. Follow up visit description and schedule

7.1. PrePex™ arm visit description

The study schedule of events is summarized in Table I. Each subject will undergo a series of study visits including the screening visit, device application visit, device and foreskin removal visit and additional follow-up visits up to 8 weeks. Below is a detailed description of the visits.

- ***Visit # 1*** – (-7) to (0) days. Screening visit will be performed to determine subject eligibility, collect medical history, current medication including pain killer's information, perform genital examination and schedule the device apply date. All these will be done after the subject has properly signed the Informed Consent Form (ICF). It is allowed to have the device applied on the same day of screening, if the subject agrees and specifically signs in the proper location in the informed consent form. The initial status of the penis and foreskin will be photographed.
- ***Visit # 2*** – Day 0. Device application visit. The subject will receive 3gr of EMLA anaesthetic cream (J&J) before the procedure starts. The subject will undergo penile measurements at flaccid or erectile status. The penis will be disinfected with Betadine, with the foreskin stretched backwards to expose the inner skin to the disinfection solution. The circumcision line will be marked with a sterile skin marker and then the operator will open the corresponding sized package of the PrePex™, and will apply the Elastic Ring to the Placement ring and place them on the penis shaft. Then, the inner ring may be rubbed with biocompatible lubricant (KY jelly). The top of the foreskin will be grasped by gloved hands and dry gauze and the foreskin will be stretched open. Then, the inner ring will be applied through the stretched top of the foreskin and over the glans penis (hereinafter, "glans") so that it will reach the glans corona (the base of the glans). At this point, the foreskin is around the inner ring and is held tight, the Elastic Ring and Placement ring is then approximated to the inner ring. The foreskin is then adjusted to fit the circumcision line to the Elastic Ring, and the Elastic Ring is rolled over the foreskin to fit firmly around the inner ring. The procedure is then completed. Then the penis will be photographed. Then, the subject's subjective feelings with the device will be recorded by means of the Visual Analogue Scale (VAS) for Pain, Discomfort, Tingling and Itching. Also, any other expected side effects and AE and change in medications will be recorded. The subject should be instructed not to touch the foreskin by himself. The subject should be instructed to return to the site with any unexpected situation, if case of pain or if the ring was moved or if the foreskin is completely detached. The subjects will be reminded to abstain from sexual intercourse and to avoid masturbation. The preparation and procedure time may be measured by stopwatch.
- ***Visit # 3-*** Day 3 following device application. In this visit the subject's genital area will be examined, subjective feelings with the device will be recorded and pain during erection by the VAS. Any side effects, AE and change in medications including use of traditional medicine will be recorded. The subject will be instructed to return to the site with any unexpected situation. The penis will be photographed. The subject will be instructed not to touch the foreskin by himself. The subject will be reminded to abstain from sexual intercourse and to avoid masturbation.
- ***Visit # 4-*** Day 7 following device application. In this visit the subject's foreskin and the PrePex™ device will be removed. The

subject’s genital will be examined and subjective feelings with the device will be recorded by the VAS. Any expected side effects and AE and change in medications will be recorded. The subject’s necrotized foreskin will be removed using sterile scissors. The Elastic Ring will be cut with a scalpel and removed. Then the Inner Ring will be removed. Then the removal area will be dressed with sterile gauze and strapped with adhesive tape. Again the subject’s genital will be examined and subjective feelings with the device will be recorded by the VAS, any expected side effects and AE and change in medications will be recorded. The subject will be instructed to return to the site with any unexpected situation. The penis will be photographed. The subject will be instructed not to touch the dressing by himself. The subject will be reminded to abstain from sexual intercourse and to avoid masturbation. The preparation and procedure time may be measured by stopwatch.

- ***Visit # 5-.*** Day 9 following device application. In this visit the dressing will be removed and the subject's genital will be examined, subjective feelings will be recorded by the VAS, and any other expected side effects and AE and change in medications will be recorded. The penis will be photographed. The subject will be instructed not to touch or rub the detached circumference wound. The subjects will be reminded to abstain from sexual intercourse until the end of the follow up.
- ***Visit # 6-.*** D14 following device application. In this visit the subject's genital will be examined, subjective penile feelings will be recorded by the VAS, and any other expected side effects and AE and change in medications will be recorded. If the penile shaft is completely healed, the subject will terminate his follow-up but should not resume sexual activity until 8 weeks following the foreskin removal. If the detached circumference wound is not healed completely, the subject will be instructed not to touch or rub the detached circumference wound. The penis will be photographed. The subject will be reminded to abstain from sexual intercourse until the end of the follow up. The subject will continue visiting on a weekly basis until 8 weeks following device removal.
- ***Potential Visit #7, #8, #9, #10, #11, #12, #13-. D21, D28, D35, D42, D49, D56, D63*** following device application. In this visit the subject's genital will be examined, subjective penile feelings will be recorded by the VAS, and any other expected side effects and AE and change in medications will be recorded. If the penile shaft is completely healed, the subject will terminate his follow-up but should not resume sexual activity until 8 weeks following the foreskin removal. If the detached circumference wound is not healed completely, the subject will be instructed not to touch or rub the detached circumference wound. The penis will be photographed. The subject will be reminded to abstain from sexual intercourse until the end of the follow up. The subject will continue visiting on a weekly basis until 8 weeks following device removal.

- ***Visit #14-.D90*** following device application. In this visit the subject's genital will be examined, subjective penile feelings will be recorded by the VAS, and any other expected side effects and AE and change in medications will be recorded. The penis will be pictured. Questionnaire regarding quality of life and satisfaction will be filled.

7.2. Surgical arm visit description

- **Visit #1: D7** folllowing surgery.
- **Subsequent visits** will be weekly (study days 14, 21, 28, 35, 42, 49, 56 and 63) for 8 weeks. In each visit the subject will be asked for any anticipated and non-anticipated adverse events or pain.

In addition, cohort participants in both arms will be scheduled for private follow-up interviews at 14 days and 3 months after their first interview in order to document and track changes in attitudes towards and feeling regarding their circumcisions, as well as their current sexual practices and satisfaction.

The circumcision procedure (both of PrePex™ arm and of Surgical arm) will be videoed for further analysis such as procedure time analysis. The penis will be pictured in each follow-up visit as well as in screening.

TABLE 1: Protocol Description and Timing of Assessments									
	Screening and Randomization Visit	Applying Visit	Physical Examination of the genital Visit	Removal Visit	Physical Examination of the genital Visit	Physical Examination of the genital Visit	Physical Examination of the genital Visit	Physical Examination of the genital Visit	Physical Examination of the genital Visit
**Visit #**	**1**	**2**	**3**	**4**	**5**	**6**	**7**	**8**	**9, 10, 11, 12, 13**
**Day (D)/week (w)**	**–7 to D0**	**D0**	**D3**	**D7**	**D9**	**D14**	**D21**	**D28**	**D35, 42, 49, 56, 63**
Informed consent	X								
Randomization	X								
Screen with Inclusion/exclusion criteria	X								
Medical history & Demography	X								
Current medication including pain killers	X	X	X	X	X	X	X	X	X
Psychosocial interview	X					X			X (D90)
Genital examination and potential pictures	X	X	X	X	X	X	X	X	X
Video Procedure		X		X					
Device apply		X							
Foreskin Removal				X					
Device Removal				X					
Subject's subjective pain, tingling and discomfort evaluation		X	X	X	X	X	X	X	X
Expected Side effects and AE		X	X	X	X	X	X	X	X
Statistical analysis of data									Interim safety report at end of the follow-up

8. Ascertainment of response variables

8.1. Training

- The PrePex™ Operators and assistants will be formally trained with the most up to date training program and certified as PrePex™ operator or assistant. The initial training will be carried out by PrePex™ Operators from Rwanda and will include, theoretical, didactic and hands on training to ensure that each operator has successfully participated in at least 10 device deployments and 10 device removals. Subsequent training programs will then be done by Zimbabwean Operators.
- All operators will also get familiarized to the Informed Consent form (ICF), Case Report Forms (CRF), and other data collection tools to be used in all phases of this study

8.2. Data collection

Data will be collected for all primary and secondary endpoints as follows:

Safety Study

8.2.1. Primary Endpoint

8.2.1.1. Primary Safety Endpoint

The primary safety endpoint is the incidence of Clinical adverse events and Device-related incidents.

- Clinical related incidents such as the following:
    - o Site bleeding (bleeding that cannot be stopped with pressure of 30 seconds and require suture)
    - o Penis diffused hematoma
    - o Penis diffused edema
    - o Incision site infection and related symptoms
- Device-related incidents
    - o Necrotic Process not initiated
    - o Device does not remain in situ for the full 7 days

Expected procedure side effects will not be included in the Primary safety endpoint, including:

- o Localized Edema
- o Ooozing
- o Clear exudates

Those expected procedure side effects will be collected separately and reported separately as a secondary endpoint

8.2.1.2. Primary Efficacy Endpoint

- Glans completely exposed (complete circumcision)

8.2.2. Secondary Endpoint

8.2.2.1. Secondary safety parameters:

- Pain assessment at key time points
    - o Subject's subjective pain, tingling, and discomfort related to the PrePex™ when applying the system, wearing it and removing it. This will be assessed by numeric pain, tingling and discomfort VAS scale

(Pain = VAS x Duration in Minutes). Measured in the first 16 hours post procedure
  - As the necrotic process initiates, it is expected that some of subjects will have temporary discomfort during the first 2 hours after deployment (hence anesthetic cream), which vanishes thereafter for the rest of the procedure period.
  - It is expected to have about 10 seconds of pain when extracting the Inner Ring during the removal procedure.
- Expected procedure side effects include:
  - Localized Edema
  - Oozing
  - Clear exudates

8.2.2.2. Secondary efficacy parameters:

- Patient satisfaction
  - Subject's subjective view regarding satisfaction during the procedure and post procedure VAS scale
- Cosmetic results
  - Objective analysis of photographs
- Time to complete healing
  - Treating physician wound status evaluation by scores of different wound parameters such as exudate type and tissue type – Granulation epithelial etc.
  - Optional validation by objective analysis of wounds by photographs
- Cost of procedure
  - Analysis of costs related to training, personnel, time, infrastructure, tools and materials
- Subject loss of work days
  - The average number of lost working hours (or potential working hours in unemployed)
- Activities of daily living restrictions
  - Listing of activities of daily living, whether they are interrupted and for how long
- Difficulties and complications during procedures
  - Operator reports
- Sexual activity
- Satisfaction by staff
- Subjects compliance to be circumcised by the PrePex™ device as a measure of the percent of subjects who agreed to participate versus the number of those offered to participate

Comparative Study

8.2.3. Primary Endpoint:

- The primary endpoint is the total operative time of the PrePex™ Device circumcision procedure versus the total operative time of surgical circumcision procedure.
  Operative Time:
  - PrePex™: Placing Placement ring on shaft till cutting of Verification Thread + First cut of dry foreskin till device removed

  - Surgical: First cut to last suture

8.2.4. Secondary Endpoints:

The two treatment arms will be compared in regards to following parameters:

- Preparation time – will be measured in addition to the primary endpoint of procedure time
  - PrePex™: Taking pants off, Draping (optional), Disinfecting (both visits), Measuring, wound Dressing
  - Surgical: Dressing for surgery, Draping (optional), Disinfecting, Anesthesia, Wound dressing
- Pain assessment at key time points
  - Subject's subjective pain, tingling, and discomfort pre during and post procedure. This will be assessed by numeric pain VAS scale combined with the duration of the pain (Pain = VAS x Duration in Minutes).
  - Measured in the first 16 hours post procedure.
- Clinical adverse event rates
- Device-related / Procedure-related incident rates
- Patient satisfaction
  - Subject's subjective view regarding satisfaction during the procedure and post procedure
- Staff satisfaction
- Cosmetic results
  - Objective analysis of photographs
- Time to complete healing
  - Treating physician objective wound status evaluation by scores of different wound parameters such as exudate type and tissue type – Granulation epithelial etc.
- Cost of procedure
  - Analysis of costs related to training, personnel, time, infrastructure, tools and materials of each of the study arms.
- Activities of daily living restrictions
  - Listing of activities of daily living, whether they are interrupted and for how long
- Sexual activity
- Subject loss of work days
  - The average number of lost working hours following each procedure (or potential working hours in unemployed subjects).
- Difficulties and complications during procedures
  - Operator reports

8.3. Quality control

Photos and data from the subject's permanent medical records (see source documentation section) will be recorded on case reports forms (CRFs). These CRFs and photos will be used to transmit the information collected in the performance of this study to the clinical database either manually or by site data entry. The CRF for this study will be of standard type with one original and a copy created, in which the original copy will be kept on site. Corresponding CRFs should be completed immediately after each subject's visit. All source data must be

typewritten or filled out in ball pens, accurately and promptly following each examination or surgery. The corresponding CRF will be completed and no fields will be left blank. CRFs entries corrections will be made only by crossing out (single line) incorrect data and writing in the revisions. All corrections must be initialed and dated by the individual performing/recording them. If the reason for the change is not obvious, an explanation will be recorded. Blacking out or using correction fluid or an eraser is not allowed to eliminate data. The investigator will review the CRFs for completeness and accuracy and must sign/date the forms where indicated. Signature stamps or substitutes are not acceptable. The investigator will retain originals of all source documents, subject consent forms, photos and study data as a permanent record. Each set of CRFs copy should be reviewed for accuracy and completion (signatures, dates, adverse events, serious adverse events, protocol departures) and maintained in the investigator's study site.

8.4. Clinical Management of Study Related Adverse Events

AEs will be clinically managed according to medical indications and the standard of care in the specific health facility. Subjects will be entitled for continuation of clinical follow-up and treatment until their study related clinical problem has been resolved, even beyond the pre-defined 8 weeks follow-up period

9. Data `Entry, Processing and Analysis

9.1. Data Entry

Data entry screens will be designed in SPSS with logic checks, skip patterns and range checks. The ZiCHIRe office will provide data management, record maintenance, data checking, cleaning, and double entry verification for all quantitative trial data collected.

9.2. Data Analysis

Safety and Efficacy Study

Baseline demographics and patient characteristics will be presented in tabular format using descriptive statistics. Binary data such as adverse events success/fail criteria will be presented as counts and percentages together with an exact 95% confidence interval. Adverse event rates will be compared descriptively with the reported rates of similar treatments. Continuous data will be represented by a mean, standard deviation, median, minimum and maximum together with 95% confidence intervals for the means.

Comparative Study

The primary end-point evaluations will be performed according to intention to treat. Categorical parameters will be summarized overall and within arms and compared by frequencies and percentage, while quantitative variables will be summarized by mean, median, standard deviation and range. In particular, the following statistical tests where applicable will be performed:

- Ordinal categorical parameters are going to be analyzed by Wilcoxon rank sum test.
- Nominal categorical parameters will be analyzed by chi-square tests/Fisher exact test (in case of low observed frequencies).
- Continuous parameters will be presented by the estimated value and calculation of 95% confidence intervals. In addition, the comparison will be by using the

relevant Student's t-test.

- Logistic regression will be used in order to compare rates adjusting for major demographic covariates.

P-values from individual statistical tests will be reported at a significance level of $\alpha=0.05$.

10. Termination policy

The study may be terminated due to any of the following reasons:

1. Device related SAEs which are life threatening in at least two cases.
2. Device failures in more than 5% of the cases.
3. Other ethical or clinical considerations
4. Decision by the investigator or DSMB that termination is in the subjects' best medical interest

10.1. Study completion

The PI will complete and report the study in satisfactory compliance with the protocol.

It is agreed that, for any reasonable cause, either the PI or the DSMB, may terminate this study, provided a written notice is submitted at a reasonable time in advance of intended termination. If the study is terminated for safety reasons, the investigator will be notified immediately by telephone, followed by written instructions for study termination notification of the Ethical Committee.

11. Protocol deviations and exceptions

The investigator should not implement any deviation from, or changes of, the protocol without agreement with the DSMB and prior review and documented approval from the Ethical Committee of an amendment, except where necessary to eliminate an immediate hazard(s) to trial subjects. The investigator should document and explain any deviation from the approved protocol and to file waivers received from the MRCZ, if applicable. The reasons for it, and, if appropriate, the proposed protocol amendments should be submitted to:

1. The DSMB for agreement
2. Ethical Committee
3. The Board
4. The regulatory authority

12. Removal of subjects from the study

A subject has the right to withdraw from the study at any time, for any reason, without prejudice to his future medical care by the physician or the institution. Should a subject (or the subject's legally authorized representative) decide to withdraw, all efforts will be made to collect and report the final visit observations as thoroughly and timely as possible. However, subject should know that if he wishes to withdraw, he will need to undergo surgical circumcision.

Subjects may be removed from the study if any one or more of the following events occur:

- Refusal of the subject to continue treatment and/or observations;
- Decision by the Investigator that termination is in the subject's best medical interest;
- Subject is lost to follow-up;

- Other ethical or clinical considerations.

13. Ethical conduct of the study

This study will be conducted in compliance with the protocol after approval of Medical Reseach Council of Zimbabwe, and according to Good Clinical Practice (GCP) and international standards such as ISO 14155. No deviation from the protocol, after MRCZ and DSMB's approval will be implemented without the prior review and approval of the Ethical Committee except where it may be necessary to eliminate an immediate hazard to a research subject. In such case, the deviation will be reported to the Ethical Committee and to the DSM Board as soon as possible.

A copy of the protocol, Informed Consent Form (ICF) and advertising material must be submitted to the Ethical Committee. Written approval of the protocol Informed Consent Form and advertising material must be obtained prior to subject enrollment by the Ethical Committee.

14. Study time schedule

ACTIVITY AREA	ACTIVITIES	Month 1				M 2	M 3	M 4	M 5
		W 1	W 2	W 3	W 4				
**Submit proposal to MRCZ**		x							
**Secure funding**		x	x	x	x				
**Training of PrePex Operators.**	Travel to Kigali by Zimbabwe Research team		x						
**Training of Research Assistants**	Familiarize with study design and procedures			x					
	Pilot tools.				x				
**Field Work**	Setting up study sites				x				
	Perform circumcisions					x	x	x	
	Subjects screening and allocation				x	x	x	x	
	Fill in CRFs					x	x	x	
	Conduct interviews for acceptability study					x	x	x	
	Follow up review visits					x	x	x	x
**Data Analysis, including regular Presentation to DSMB**	Review of filled CRFs and questionnaires					x	x	x	
	Data cleaning and Entry							x	x
	Analysis report to DSMB					x	x	x	x
**Steering Committee Meetings**	Hold monthly steering committee meeting				X	x	x	x	x
**Report Writing**	Write preliminary report.								x
	Share report.								X

D. Organization

1. Participating Investigators

- Dr Gerald Gwinji**,** MBChB, MPH. PS- MoHCW, Principal Investigator
- Prof Mufuta Tshimanga, MD; Public Health Physician, Department of Community Medicine, University of Zimbabwe. Co-Principal Investigator
- Dr. Mangwiro,MMed Urology, Department of Surgery, University of Zimbabwe
- Dr Owen Mugurungi, MBChB, Msc; Director AIDS- TB , MoHCW
- Dr Munyaradzi Murwira, MBChB, MPH. Director ZNFPC
- Dr. Danuta Kasprzyk,PhD, Research Leader, Battelle
- Dr. Daniel Montaño, PhD, Research Leader, Battelle
- Mrs. Daisy Nyamukapa, Program Officer, UNFPA
- Mr. Sinokuthemba Xaba, MC Program Officer, AIDS-TB

2. Data Coordinating Center

ZiCHiRe DE Unit. This unit is headed by a data analyst, Mrs. Patricia Gundidza, an experienced statistician. Data will be cleaned, entered, analyzed and safely stored here.

3. Study administration

3.1. Steering committee

The PrePex Study Steering Committee (PSSC) is vested with primary responsibility for the ethical running of the trial. It is responsible for assisting and guiding the research team. It also monitors the study implementation and budget. The PSSC will operate in an advisory capacity leaving the principal investigator to implement the study procedures (See Annex VI).

3.2. *Data* Safety *Monitoring Board*

A data safety and monitoring board will be established to monitor the study conductance. The committee might include the following personnel

Name	Affiliation
Prof G. Muguti	Professorial Chair Of Surgery University of Zimbabwe
Prof. Z. Chirenje	University of Zimbabwe
Robert Ntozini	Trial Bio statistician- Zvitambo
Frances Cowan	ZAPP
Mr Gunda	MRCZ
Helen Wayce	

3.3. Study Monitoring

Study Records/Source Document Inspection

The investigator will allow the research team (nurses, data managers etc.), representatives of Sponsor, its monitoring team, the MOHCW of Zimbabwe, and other governmental regulatory agencies to monitor/audit/inspect all study records, CRFs, IRB/IEC records, and corresponding portions of the subject's office and/or hospital medical records at regular intervals throughout the study. These monitoring/audits/inspections are conducted to verify adherence to the protocol, integrity of the data being captured on the CRFs and compliance with applicable regulations. Study reports will not identify subjects by name or other identifiers.

Monitors

Each study's site will be monitored by a qualified representative of MRCZ or any qualified monitor delegated to do so, on behalf of MRCZ to monitor each study subject's data and study conduct at regular intervals throughout the course of the study according to a pre-defined monitoring plan. On site monitoring of the investigator's facilities aids in ensuring compliance with the protocol.

Any deficiency noted during the monitoring visits will be discussed with the investigator and the corrective actions to be taken agreed upon. Should the MRCZ determine, at any time during the study that the investigator is not in regulatory and/or protocol compliance, measures necessary to establish compliance will be implemented. If compliance cannot be maintained, the MRCZ will suspend or terminate the study at this site.

Monitoring Plan

I. Pre-study visit

1. Assess the site's infrastructure (staff and facility) for the capability to conduct the study.
2. Evaluate Investigator and staff's Experience, Qualifications and Capabilities- signed and dated CVs.
3. Financial Disclosure information. The MRCZ may waive the pre-study visit in certain sites based on previous knowledge or experience with the site or personnel, or other reasons as deemed acceptable.

II. Study Initiation Training

Orients the investigator and staff involved in the study on

1. Protocol content and procedures
2. CRF and fill-in process including queries resolution process
3. GCP and other regulatory requirements
4. Informed Consent form and process
5. AE, SAE, Safety reporting
6. Ethical Committee
7. Investigational product accountability
8. Subject information- subject's identification log, subject pre-screening and screening logs, subject enrollment log, subject study visit log
9. Study monitoring
10. Investigator's site file (ISF)
11. Report of AE back from the MRCZ to the sites (Expedited Safety reports)

12. Expectations from the site regarding data collection and timelines
13. Ministry of health

III. Regular Monitoring visits

1. Check on the progress of the study
2. Protocol and GCP compliance
3. Informed Consent form and process
4. CRF completion, correction, source data verification
5. AE, SAE, Safety reporting
6. Investigational product
7. Investigator's study file
8. Pictures and videos

Detailed monitoring schedule to be provided at study initiation

IV. Final Study visit - Close out monitoring visit

Ensure Investigator understands the on-going responsibilities

1. Record archival practices for source documents and CRFs after completion of the study - up to 5 years.
2. Follow-up of on-going device related adverse events - up to 30 days after completion of the study and of SAE – until resolution.
3. Finalizing all "open" issues and complete Source Data Verification (SDV).

E. Funding organizations

1. UNFPA
2. BATTELLE
3. MOHCW

ANNEX I

Sampling Instructions

1. Clients will be counseled and tested.
2. If the male client is negative, he is told about MC.
3. If he accepts he is also told about the study
4. If he accepts, screening is done using the inclusion criteria
5. If he is eligible then, the informed consent is offered and explained.
6. He will then be allocated to a trial arm (A bag with numbered marbles from 1-120 for the device and 1-60 for surgical is raised for the client to pick one). The number will determine which method he will be offered.
7. If the client does not meet the inclusion criteria, then he is offered the usual surgical MC method.

(N.B Numbers on the marbles will be determined by the number of health facilities where clients are going to be found- we will use proportionate sampling for the numbers. i.e. for health facilities where more boys of the age 18-30 go for MC, we will get more numbers for the sample.)

ANNEX II

Informed Consent Procedure

- In obtaining and documenting informed consent, the investigator should comply with the applicable regulatory requirements, and should adhere to GCP (Good Clinical Practice), HIPAA (Health Insurance Portability and Accountability Act) and the ethical principles that have their origin in the Declaration of Helsinki
- Prior to the beginning of the trial, the investigator should have the written informed consent form and any other written information approved by the MRCZ before it is provided to subjects
- The written informed consent form and any other written information to be provided to subjects should be revised whenever important new information becomes available that may be relevant to the subject's consent. Any revised written informed consent form, and written information must be approved by the MRCZ before it is made available for subject signature. The subject or the subject's legally acceptable representative should be informed in a timely manner if new information becomes available that may be relevant to the subject's willingness to continue participation in the trial. The communication of this information should be documented
- Neither the investigator, nor the trial staff, should coerce or unduly influence a subject to participate or to continue to participate in a trial. He will also testify of his agreement by a signature on the same ICF
- The investigator, or a person designated by the investigator, should fully inform the subject or, if the subject is unable to provide informed consent, the subject's legally acceptable representative, of all pertinent aspects of the trial including the Ethical committee approved written informed consent
- Before informed consent may be obtained, the investigator, or a person designated by the investigator, should provide the subject or the subject's legally acceptable representative ample time and opportunity to inquire about details of the trial and to decide whether or not to participate in the trial. All questions about the trial should be answered to the satisfaction of the subject or the subject's legally acceptable representative.
- Prior to a subject's participation in the trial, the Ethical Committee approved written informed consent form should be signed and personally dated by the subject or by the subject's legally acceptable representative, and by the person who is authorized to conduct the informed consent discussion
- Prior to participation in the trial, the subject or the subject's legally acceptable representative should receive a copy of the signed and dated written informed consent form and any other written information provided to the subjects. During a subject's participation in the trial, the subject or the subject's legally

acceptable representative should receive a copy of the signed and dated consent form updates and a copy of any amendments to the written information provided to subjects

- Subject or the subject's legally acceptable representative must provide informed consent and sign and date with his hand writing an Informed Consent Form (ICF) prior to any study related procedures being performed. The person reviewing the ICF with the subject must also sign and date the ICF. Each of the signers must sign and date the Form in the presence of the other signer.

If subject or the subject's legally acceptable representative is unable to comprehend and sign the Consent Form, subject must not be enrolled in the study.

ANNEX II

Guidelines for Photography

Photos:

Photos of the Penis should be taken in each study visit according to the following guidelines:

1. The Camera must have a memory card sufficient to contain 4334 expected photos of the largest file produced by the camera (1210+10% photos of surgical arm and 2730+10% photos of PrePex™ arm).
2. A spare set of new camera batteries must always be kept in the camera pack.
3. A spare camera memory card must always be kept in the camera pack.
4. Minimum of 2 photos, 1 from top and 1 from bottom of the glans must be taken presenting 360˚ around the glans and showing the frenulum. It is recommended to take 4 photos from 4 directions around the glans
5. Photos will be taken while a special photo frame is placed around the penis including the subject study number, date of photo, study day, and VAS pain value.
6. Photos must be taken with a predefined Digital Camera
7. Camera settings must be on highest definition and resolution
8. Photos must be taken from a fixed distance with predefined lighting
9. Before releasing a subject photos must be verified for quality, specifically –
    1. Focus of the photo on the skin surface
    2. Lighting
    3. Inclusion of subjects data in the photo

At the end of each photo day, all photos must be downloaded to a PC saved in a specific location and backed up on a removable Hard Drive. The Removable Hard Drive must be kept separate from the PC for ensuring no loss of data. Photos must never be erased from the camera digital memory card. In case the card is full a new card will be used and the full card will be saved in a secured location.

Video

Video of each circumcision procedure may be done for each subject, the video will be used for analysis of the exact procedure time. The video must be performed according to the following guidelines:

10. The video must record all time without pausing or stopping the recording in any case, even if moving the camera around. This is important to ensure there is no loss of exact timing of the procedure

11. The Video recording should start when the subject enters the room and stop when the subject leaves the room

12. The video should not include the subject face, however if the subject face must be included to better capture the whole procedure, it will be later blurred.

13. The video must show the different procedures being done in each phase of the circumcision procedure

14. For the PrePex™ study arm, the video should be taken during the deployment procedure visit and during the removal procedure visit

15. The video must include a capture of the subject photo frame including the subject study number and date

16. The videographer must ensure he has recording tapes (or other memory equipment) sufficient for 8 hours of video for each video day

17. The videographer must ensure he has power supply for the Video camera for 8 hours of constant video.

18. At the end of each video day 2 copies of the video taken must be kept on separate secured locations, marked with the video date.

ANNEX III

INVESTIGATOR AGREEMENT FOR PROTOCOL

I have read the foregoing protocol" **Safety and efficacy study of the** PrePex™ **circumcision device for rapid scale up of adult male circumcision programs**", and agree to:

- Conduct the study as outlined herein;
- Maintain the confidentiality of all information received or developed in connection with this protocol and
- Conduct this study in accordance with GCP Standards and any other applicable local/state laws and regulations;
- Comply with the signed investigators agreement.

________________________________ _________________________

Investigator Signature **Date**

____

Investigator name in capital letters

____

Telephone number:

____

Fax number:

____

Email address:

ANNEX IV

RECORDS HANDLING AND KEEPING

Source Documents

Source documents are the initial documents whereon subject data are recorded. This includes, but is not limited to, original subject files, hospital records, and original recordings/tracings from automated instruments, etc.

The MRCZ allows to se dedicated work sheets to serve as source documents to collect the available source data, on top of all other applicable source documents in the site.

All information captured on the CRF should be accurately supported by the source documents unless specifically approved and documented by MRCZ.

For example, each subject's source documents should include (but not be limited to):

- Documenting the Informed consent process
- Subject full name and identification
- Date of each study required visit with a description of the visit and the results of each procedure that was performed
- A full and comprehensive anamnesis that will cover subject's medical history, current disease etc.
- All concomitant procedures and medications for the screening eligibility purposes and regular visits

Any additional information relevant to the study should be included in the subject's source documents. In particular, any deviations from the study protocol or procedures should be recorded in the source documents, if noted. For example, if study required procedures or visits are not completed or are completed outside the time frame specified in the protocol, the reasons for the departure should be explained in the source documents, or mentioned whether it was waived by the MRCZ in advance. The investigator must maintain all study documentation at least 2 years after the last approval of marketing application and until there are no pending or contemplated marketing application or at least 2 years have elapsed since the formal discontinuation of clinical development of the investigational product. These documents should be retained for a longer period, however, if required by the applicable regulatory requirements or by an agreement with the MRCZ. The MRCZ should inform the investigator in writing when the trial-related records are no longer needed.

ANNEX V

REGULATORY OBLIGATIONS

Sponsor's Obligations

Clinical research studies are subject to the Zimbabwe National Ethical Committee approval. A Sponsor must assume the following responsibilities and must keep the required records. He/she must:

1. Provide the Investigator with the necessary information- Protocol and Device User's Manual or Instructions for Use (maybe incorporated to the Investigator Brochure).
2. Inform the Investigator of all new information that may affect his/her decision of whether to continue their participation in the study.
3. Provide the supplies (PrePex device) for the investigation.
4. Provide all study related tools and materials
5. Provide training

<u>Maintain the following records:</u>

1. Signed Investigator Agreement
2. Records of device shipment and disposal (shipping receipts, material destruction records, etc.)
3. Other records as required by the Health Authority.

Investigator's Obligations

Clinical research studies are subject to the regulations of the Regulatory Authority of the country. Upon signing the protocol, the Investigator agrees to assume the following responsibilities and to keep the required records for a period of three years following completion of the study and to file the required reports in a timely manner:

1. Conduct the Investigation in compliance with the protocol. Changes to the protocol may only be made after approval by the DSMB and the Ethical Committee, or when necessary to protect the safety, rights or welfare of a subject.
2. Personally conduct or supervise the investigation.
3. Read and understand the information in the Protocol and Investigator Brochure.
4. Be aware of the potential risks and side effects of the PrePex device.
5. Ensure that all associates, colleagues and employees assisting in the conduct of the study are informed about their obligations.

6. Inform all subjects that the device is being used for investigational purposes and ensure that the requirements related to obtaining the informed consent are met.

   Maintain the following records (for a period of three years following completion of the study):

1. Signed copy of the protocol.
2. Signed consent forms
3. All correspondence as relates to the clinical trial
4. Case Report Forms

File the following reports:

1. Serious unexpected adverse device related effects reports received by the MRCZ.
2. Regular Serious Adverse Event reports produced by the PI.
3. Deviations, which were made from the protocol for emergency use, must be reported to the MRCZ as soon as possible, but no later than five working days after its occurrence.

ANNEX VI

PrePex Study Steering Committee - Terms of Reference (TOR)

1. Main Authority

The PrePex Study Steering Committee (PSSC) will be chaired by an individual who is independent of the investigators who designed and are implementing the trial, in order to provide independent oversight of the study.

The Steering Committee will have other independent representation, including statistical expertise, and lay individuals from either the general public or interest groups. Trial investigators will also sit on this committee. The Study Steering Committee (PSSC) provides overall supervision of the trial on behalf of the Ministry of Health & Child Welfare (MOH&CW) and the Medical Research Council of Zimbabwe (MRCZ).

2. Main Priority and Objectives of Committee

The Study Steering Committee is vested with primary responsibility for the ethical running of the trial. It is responsible for assisting and guiding the research team. It also monitors the study implementation and budget control.

The group should meet initially to finalize the protocol and organization. Once the trial is running the group should meet regularly to discuss and review trial issues.

The Study Steering Committee should operate in an advisory capacity leaving the principal investigator to implement any decisions.

3. Main Duties and Responsibilities

- Monitor and supervise progress of study implementation
- Discuss Study Protocol (deviations and possible amendments)
- Review Interim Results
- Consider recommendations of the DMB
- Advocacy and communication issues
- Resource mobilization
- Budgetary controls

4. Constitution

Meetings

- Meetings will be held monthly

Chairperson and Secretariat

- Chairperson will be MOHCW and co-chairperson will be UZ/WHO/CSO.
- Secretariat will be AIDS & TB Unit/CSO

Membership

1. MOHCW
2. WHO
3. MRCZ
4. National AIDS Council
5. USAID
6. PSI
7. UNFPA
8. ZNFPC
9. SAYWHAT
10. Battelle Memorial Institute

11. Biostatistician from Zvitambo
12. UNAIDS- Helen Jackson

Quorum

- Two-thirds of members constitute a quorum

Standard Agenda Items

- Apologies
- Minutes of previous Meeting and Matters Arising
- Progress Update
- Budget Review
- AOB
- Date of next meeting

Members Responsibilities

- Active participation in deliberations
- Advocacy work for the study

ANNEX VII -. REFERENCES

Articles from journals and medical publications including WHO

Anonymous Newer approaches to HIV prevention*. Lancet* 2007;369(9562):615 (Editorial).

Auvert B, Taljaard D, Lagarde E, Sobngwi-Tambekou J, Sitta R, Puren A. Randomized, controlled intervention trial of male circumcision for reduction of HIV infection risk: the ANRS 1265 Trial. *Public Library of Science Medicine* 2005;2(11) :e298. Erratum in: *PLoS Med*. 2006;3(5):e298.

Bailey R, Moses S, Parker CB, et al. Male circumcision for HIV prevention in Kisumu, Kenya: A randomized controlled trial. *Lance*t 2007;369:643-56.

Bailey RC. *Evaluation of a trial intervention integrating male hygiene and circumcision with reproductive health in Nyanza Province.* Unpublished report to AIDSMARK; 2002.

Bailey RC. *Needs assessment: Introduction of male circumcision services for HIV prevention in Kisumu and Suba districts. Kenya, funded by US CDC (Kenya), PEPFAR and CHF International*. 2006.

Bailey RC, Egesah O, Rosenberg S. Male circumcision for HIV prevention: a prospective study of complications in clinical and traditional settings in Bungoma, Kenya. *Bull WHO* 2008;9:669-77.

Bailey RC, Muga R, Poulussen R, Abicht H. The acceptability of male circumcision to reduce HIV infections in Nyanza Province, Kenya. *AIDS Care* 2002;14(1):27-40.

Bailey RC, Neema S, Othieno R. Sexual behaviors and other HIV risk factors in circumcised and uncircumcised men in Uganda. *Journal of Acquired Immune Deficiency Syndromes* 1999;22(3):294-301.

Gray RH, Kigozi G, Serwadda D, et al. Male circumcision for HIV prevention in men in Rakai, Uganda: a randomised trial. *Lancet* 2007;369(9562):657-66.

Halperin DT, Fritz K, McFarland W, Woelk G. Acceptability of adult male circumcision for sexually transmitted disease and HIV prevention in Zimbabwe. *Sexually Transmitted Diseases* 2005;32(4):238-9.
Kahn JG, Marseille E, Auvert B. Cost-effectiveness of male circumcision for HIV prevention in a South African setting. *Public Library of Science Medicine* 2006;3(12):e517.

Kebaabetswe P, Lockman S, Mogwe S, Mandevu R, Thior I, Essex M, Shapiro RL. Male circumcision: an acceptable strategy for HIV prevention in Botswana. *Sexually Transmitted Infections* 2003;79(3):214-9.

Kigozi G, Watya S, Polis CB, et al. The effect of male circumcision on sexual satisfaction and function, results from a randomized trial of male circumcision for human immunodeficiency virus prevention, Rakai Uganda. *Brit J Urol Int* 2008;101:65-70.

Lagarde E, Dirk T, Puren A, Reathe RT, Bertran A. Acceptability of male circumcision as a tool for preventing HIV infection in a highly infected community in South Africa. *AIDS* 2003;17(1):89-95.

Marck J. Aspects of male circumcision in sub-equatorial African culture history. *Health Transition Review* 1997;7(Suppl):337-60.

Mattson CL, Bailey RC, Muga R, Poulussen R, Onyango T. Acceptability of male circumcision and predictors of circumcision preference among men and women in Nyanza Province, Kenya. *AIDS Care* 2005;17(2):182-94.

Moses S, Bradley JE, Nagelkerke NJ, Ronald AR, Ndinya-Achola JO, Plummer FA. Geographical patterns of male circumcision practices in Africa: association with HIV seroprevalence. *International Journal of Epidemiology* 1990;19(3):693-7.

Ngalande RC, Levy J, Kapondo CP, Bailey RC. Acceptability of male circumcision for prevention of HIV infection in Malawi. *AIDS and Behavior* 2006;10(4):377-85.

Nnko S, Washija R, Urassa M, Boerma JT. Dynamics of male circumcision practices in northwest Tanzania. *Sexually Transmitted Diseases* 2001;28(4):214-8.

Rain-Taljaard RC, Lagarde E, Taljaard DJ, Campbell C, MacPhail C, Williams B, Auvert B. Potential for an intervention based on male circumcision in a South African town with high levels of HIV infection. *AIDS Care* 2003;15(3):315-27.

Scott BE, Weiss HA, Viljoen JI. The acceptability of male circumcision as an HIV intervention among a rural Zulu population, Kwazulu-Natal, South Africa. *AIDS Care* 2005;17(3):304-13.

UNAIDS. *Collaborating with traditional healers for HIV prevention and care in sub-Saharan Africa: suggestions for program managers and field workers*. Geneva: Joint United Nations Programme on HIV/AIDS; 2006 (Best Practice Collection UNAIDS06.28E / ISBN 92-9-173342-3). (http://data.unaids.org/Publications/IRC-pub07/JC967-TradHealers_en.pdf, accessed 27 February 2007).

van Dam J, Anastasi M-C. *Male circumcision and HIV prevention: directions for future research.* Washington DC: Population Council; 2000 (Horizons Report). (http://www.popcouncil.org/pdfs/circumcision.pdf, accessed 27 February 2007).

Weis HA, Halperin D, Bailey RC, Hayes RJ, Schmid G, Hankins CA. Male circumcision for HIV prevention: From evidence to action? *AIDS* 2008;22:567-78.

Westercamp N, Bailey RC. Acceptability of male circumcision for prevention of HIV/AIDS in sub-Saharan Africa: A Review. *AIDS and Behavior* 2007;11(3):341-55.

Williams BG, Lloyd-Smith JO, Gouws E, Hankins C, Getz WM, Hargrove J, de Zoysa I, Dye C, Auvert B. The potential impact of male circumcision on HIV in sub-Saharan Africa. Public Library of Science Medicine 2006;3(7):e262.

Male circumcision for HIV prevention publications in chronological order for the past 3 years

1. Country experiences in the scale-up of male circumcision in the Eastern and Southern Africa region: two years and counting. Meeting report from a sub-regional consultation Windhoek, Namibia 9-10 June 200923 July 2009
2. Male circumcision situation analysis toolkit. 1 May 2009
3. Operational guidance for scaling up male circumcision services for HIV prevention. WHO and UNAIDS. 16 January 2009
4. Male circumcision quality assessment toolkit. A toolkit to assess the safety and quality of services. 14 January 2009
5. Male circumcision quality assurance guide. A guide to enhancing the safety and quality of services. 30 October 2008
6. Safe, voluntary, informed male circumcision and comprehensive HIV prevention programming. Guidance for decision-makers on human rights, ethical and legal considerations. 16 September 2008
7. Consultation on male circumcision and HIV prevention in the African Region. Meeting report, Brazzaville, Congo, 2-4 April 2008. 2 April 2008
8. Male circumcision and HIV prevention in Eastern and Southern Africa : communications guidance. 16 March 2008
9. Manual for male circumcision under local anaesthesia. WHO | UNAIDS | JHPIEGO | Version 2.5C. 29 January 2008.
10. Male circumcision information package. 15 December 2007
11. Male circumcision: global trends and determinants of prevalence, safety and acceptability. 14 December 2007
12. Male circumcision quality assurance guidance expert review meeting. Meeting report, Montreux, Switzerland, 12-13 November 2007 12 November 2007
13. East and Southern Africa faith-based organizations: Male circumcision consultation . Meeting report, Limuru, Kenya, 20-21 September 2007
14. Male circumcision and HIV prevention: operations research implications. International consultation report | Nairobi, Kenya, 21- 22 June 2007
15. Countries in the Eastern and Southern Africa region agree to accelerate scale up of male circumcision services in the context of HIV prevention. Meeting report, Harare, 7-9 may, 2007
16. New data on male circumcision and HIV prevention: Policy and programme implications. 6 March 2007
17. Social science perspectives on male circumcision for HIV prevention. Meeting report, Durban, 18-19 January, 2007
18. Strategies and approaches for male circumcision programming. Meeting Report, 5 - 6 December 2006, Geneva
19. Regional consultation on safe male circumcision and HIV prevention. Meeting report, Nairobi, Kenya, 20-21 November, 2006
20. Male circumcision: Africa's unprecedented opportunity. 14 November 2006
21. The male circumcision and HIV prevention country consultation meeting. Meeting report, Swaziland, September 26-27 2006
22. Male circumcision and HIV prevention Tanzania country stakeholder consultation. Meeting report, Tanzania, 14-15 September 2006
23. Male circumcision consultative meeting. Meeting report, Lusaka, Zambia, 11-12 September 2006

24. Kenya stakeholder consultation on male circumcision in the context of HIV prevention. Meeting report, Kenya, 6 September 2006
25. Male circumcision and HIV prevention country consultation meetings. Meeting report, Lesotho, 25 July 2006
